# Epigenetic modifier gene mutations in chronic myeloid leukemia (CML) at diagnosis are associated with risk of relapse upon treatment discontinuation

**DOI:** 10.1038/s41408-022-00667-9

**Published:** 2022-04-20

**Authors:** Shady Adnan Awad, Oscar Brück, Naranie Shanmuganathan, Timo Jarvinen, Hanna Lähteenmäki, Jay Klievink, Hazem Ibrahim, Soili Kytölä, Perttu Koskenvesa, Timothy P. Hughes, Susan Branford, Matti Kankainen, Satu Mustjoki

**Affiliations:** 1 Hematology Research Unit Helsinki, University of Helsinki and Helsinki University Hospital Comprehensive Cancer Center, Helsinki, Finland; 2 Translational Immunology Research Program and Department of Clinical Chemistry and Hematology, University of Helsinki, Helsinki, Finland; 3 iCAN Digital Precision Cancer Medicine Flagship, Helsinki, Finland; 4 Clinical Pathology Department, National Cancer Institute, Cairo University, Giza, Egypt; 5 Department of Haematology, Royal Adelaide Hospital and SA Pathology, Adelaide, SA Australia; 6 Department of Genetics and Molecular Pathology and Centre for Cancer Biology, SA Pathology, Adelaide, SA Australia; 7 Precision Medicine Theme, South Australian Health and Medical Research Institute (SAHMRI), Adelaide, SA Australia; 8 School of Pharmacy and Medical Science, University of South Australia, Adelaide, SA Australia; 9 School of Medicine, University of Adelaide, Adelaide, SA Australia; 10 Stem Cells and Metabolism Research Program, Faculty of Medicine, University of Helsinki, Helsinki, Finland; 11 HUS Diagnostic Center, HUSLAB, Helsinki University Hospital, University of Helsinki, Helsinki, Finland

**Keywords:** Genetics research, Chronic myeloid leukaemia, Risk factors, Innate immunity

**TO THE EDITOR:**

The implementation of tyrosine kinase inhibitors (TKIs) has dramatically improved the outcome of CML patients with the overall survival approaching general population [1]. Hence, the achievement of durable treatment-free remission (TFR) after TKI discontinuation has emerged as a new treatment goal [2]. In several studies, approximately half of CML patients in deep molecular remission (DMR) can successfully maintain TFR after TKI discontinuation [3]. Factors such as the duration of TKI treatment and DMR prior to TKI stop, the 3 month halving time, as well as the number and function of immune cells, namely NK cells, have been suggested to affect and predict the outcome of TKI discontinuation [4–6].

Several recent studies have reported the association of somatic mutations involving cancer-associated genes with treatment outcome in chronic phase (CP) CML [7–10]. Mutations in epigenetic modifier genes, such as *ASXL1* and *DNMT3A*, represent the major fraction of mutations in CP-CML [11]. Despite the epigenetic modifier gene mutations being reported to associate with inferior responses to TKI therapy [12], such mutations can also be found in patients achieving DMR [7, 8] who would be eligible for attempting TKI discontinuation. The role of somatic mutations in predicting TFR has not been explored.

To investigate the potential effect of epigenetic modifier gene variants on the outcome of TKI discontinuation, we analyzed diagnostic samples from 47 CML patients who have attempted TKI discontinuation using targeted sequencing of selected cancer-associated genes. Our cohorts included 32 patients from the Helsinki University Hospital (cohort 1) and 15 patients monitored in South Australia (cohort 2). Only the availability of sample from the time of diagnosis and later TKI discontinuation attempt were used as selection criteria for the study. Loss of major molecular response was encountered in 30 of 47 (64%) patients after TKI discontinuation, while 17 of 47 (36%) patients maintained TFR. The median follow-up time of patients who maintained TFR is 71 months (12–138 months). Diagnostic samples were selected for mutation profiling to enable the identification of somatic cancer-associated mutations in CML leukemic cells, as at the time of TKI discontinuation after sustained DMR, patients typically have undetectable amount of leukemia cells left. Details on patient characteristics and experimental methods are included in the Supplemental Tables 1,2 and Supplemental materials.

Overall, we identified cancer-associated mutations in 12 of 47 patients (26%) (Table 1), consistent with previous studies reporting the presence of mutations to be 20% in CP patients with optimal response to TKIs [7–10]. The majority of variants was identified in diagnostic samples from patients who relapsed after TKI discontinuation (10/30 patients, 33%) compared to patients maintaining TFR (2/17 patients, 12%, *p* = *0.052)* (Fig. 1a and Supplemental Fig. 1). Mutations in the epigenetic modifier genes were the most common mutation type (9/12, 75%). *ASXL1* was the most frequently mutated gene in 5 patients (Table 1). Other mutated epigenetic modifier genes included *KDM6A* and *DNMT3A*. Overall, mutations in epigenetic modifier genes were more frequently encountered in patients who relapsed after TKI discontinuation (8/30 patients, 27%) compared to patients who maintained TFR (1/17 patients, 6%), *p* = *0.041* (Fig. 1b and Supplemental Fig. 1).

**Table 1:** Cancer-related gene mutations in CML patients attempting TKI discontinuation.

Patient	Outcome	Variant	Gene	Predicted effect	AA Change	VAF
pt_1	relapse	20-32434638-AG-A	*ASXL1*^a^	frameshift deletion	p.G643fs	5%
pt_2	relapse	X-45079223-C-T	*KDM6A*^a^	nonsense	p.Q1058X	6%
pt_4	relapse	7-152177073-G-A	*KMT2C*^a^	missense	p.P2794S	44%
pt_5	relapse	12-49024702-C-T	*KMT2D*^a^	missense	p.G5310R	27%
pt_9	relapse	1-85270827-AT-A	*BCL10*	frameshift deletion	p.I46fs	7%
pt_12	relapse	9-77922307-T-G	*GNAQ*	missense	p.M59L	6%
pt_33	relapse	20-32435461-G-T	*ASXL1*^a^	nonsense	p.E917*	50%
pt_34	relapse	20-32434789-C-T	*ASXL1*^a^	nonsense	p.R693*	39%
pt_35	relapse	20-32436404-CCA-A	*ASXL1*^a^	frameshift deletion	p.S1231fs	7%
pt_36	relapse	2-25243930-G-A	*DNMT3A*^a^	missense	p.R635Q	43%
pt_22	TFR	20-32434638-A-AG	*ASXL1*^a^	frameshift insertion	p.G642fs	31%
pt_47	TFR	21-34880643-C-A	*RUNX1*	nonsense	p.S141*	17%

Outcome: outcome of TKI discontinuation, *AA* amino acid, *VAF* variant allele frequency.

^a^indicates epigenetic modifier genes.

**Fig. 1:**
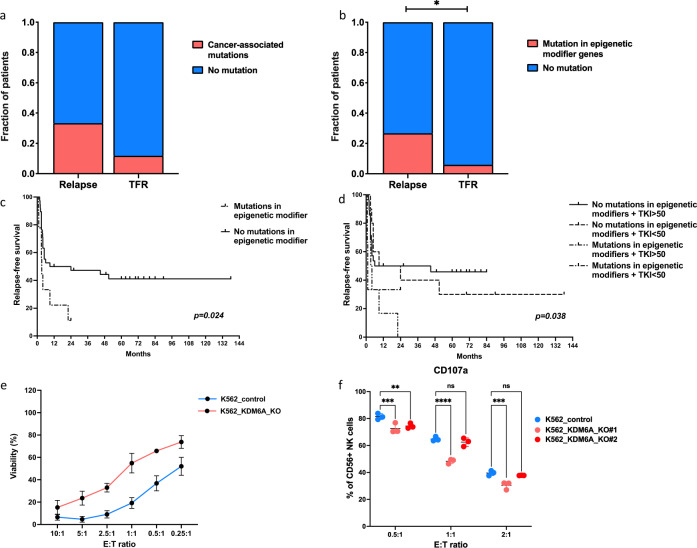
The impact of epigenetic modifier gene mutations on the outcome of TKI discontinuation. Stacked columns comparing the prevalence of mutations in **a** cancer-associated genes and **b** epigenetic modifier genes between relapse and TFR patient groups (*n* = 47, 30 relapse and 17 TFR patients). Cancer-associated mutations were identified in 10/30 relapse patients compared to 2/17 TFR patients. Epigenetic modifier gene mutations were identified in 8/30 relapse patient compared to 1/17 TFR patients. The list of the identified mutations is provided in Table 1. Survival curves comparing the relapse-free survival rates of patients (*n* = 47) classified according to **c** the presence of epigenetic modifier gene mutations using log-rank test, **d** combinations of epigenetic modifier mutations and duration of TKI treatment prior to stop using stratified log-rank test. Scatter plots showing **e** the sensitivity of *K562-KDM6A-KO* cells to NK cells (expanded from freshly isolated NK cells from two different healthy donors’ buffy coats) induced cytotoxicity compared to control K562 cells, **f** Expression of CD107a degranulation marker on CD56 + NK cells cocultured with either K562_control or K562_KDM6A_KO cells (clones #1 and #2). (*) *p* < *0.05*, Chi-square test.

Univariable and multivariable logistic regression analyses were performed for predictive factors of TFR (variables used in the analyses are listed in the Supplemental Table 3). Immunological parameters were also included from the Euro-Ski patients [5] in cohort 1. The presence of an epigenetic modifier gene variant and the duration of TKI treatment prior to stop were predictive of the TKI stop outcome in the univariable analysis (Supplemental Table 3). Patients with mutations in epigenetic modifier genes had worse relapse-free survival (RFS) rates compared to patients without mutations (median RFS 3.2 and 16.5 months respectively, *p* = *0.024*, hazard ratio = 3.55, 95% CI: 1.18–10.69) (Fig. 1c). We also performed multivariable analysis, but only borderline significant values were observed for the on-TKI duration and presence of mutations at diagnosis (Supplemental Table 3). Integration of the on-TKI duration to the epigenetic mutation slightly improved the separation of groups, but the presence of the epigenetic mutations seemed to be the strongest predictor of relapse after TKI discontinuation (Fig. 1d).

*ASXL1* was the only recurrently mutated gene in our study, and it was detected in one patient who maintained TFR and four relapse patients. This is in agreement with the reported controversial prognostic value of *ASXL1* mutations in CP-CML [12]. Variants in other epigenetic modifier genes, such as *KDM6A*, were only identified in relapse patients. Recurrent *KDM6A* mutations have been previously reported in CP-CML [7]. *KDM6A* is a histone lysine demethylase and a tumor suppressor, that is recurrently mutated in AML (acute myeloid leukemia) [13], multiple myeloma (MM) [14], and solid tumors [15]. *KDM6A* is a key regulator of the development and the phenotype of various immune cells, such as NK [16], NKT [17], and T cells [18]. *KDM6A* has been reported to modulate immune surveillance in MM, via the control of expression of major histocompatibility complex I and II molecules [19]. Furthermore, *KDM6A* deletions in medulloblastoma have been shown to impair immune cells recruitment [15].

Given the suggested important role of immune responses in TFR and the reported role of *KDM6A* in modulating tumor immune responses, we next investigated the functional consequences of *KDM6A* mutations in CML cells as an example of relapse-associated epigenetic modifier mutation. At first, we sorted granulocyte, T-, NK-, and NKT-cell populations from diagnosis and remission samples from the patient who relapsed after TKI discontinuation and had a somatic stop-gain *KDM6A* mutation. Deep amplicon sequencing was used to detect *KDM6A* mutation from the sorted fractions, and interestingly, in the diagnostic samples we identified *KDM6A* mutation to be present in both granulocyte and NK-cell populations at a comparable VAF (8% and 6%, respectively), but not in the T- or NKT-cell populations. The mutation was not found in any population in the remission samples, suggesting the leukemic origin of the mutation, and that *KDM6A*-mutated NK cells at diagnosis were part of the malignant clone. This was confirmed by the detection of BCR-ABL hybrid in NK cells at diagnosis (Supplemental Fig. 3a). Next, we wanted to investigate the potential effect of *KDM6A* mutations on the immune interactions between CML and NK cells. Using CRISPR/Cas9 gene editing, we introduced knockout (KO) of *KDM6A* gene in K562 CML cells. While the effects of KDM6A-KO were notable on the status of histone acetylation and methylation in K562 cells, there was no change in the sensitivity of K562 cells to TKIs (Supplemental Fig. 3b, c). Interestingly, K562-*KDM6A*-KO cells exhibited reduced sensitivity to NK-cell mediated cytotoxicity at different E:T ratios, compared to control cells (*p* < 0.001). The reduced sensitivity of K562-*KDM6A*-KO cells was preserved using either expanded/activated NK cells or freshly isolated NK cells from healthy donors (Fig. 1e, Supplemental Fig. 3d, e). In accordance, NK cells showed reduced surface expression of CD107a (degranulation marker) when cocultured with K562-*KDM6A*-KO cells compared to coculture with control cells (Fig. 1f). In contrast, knockout of *KDM6A* in the cytotoxic NK-cell line, NK-92, was not associated with reduced cytotoxic activity against K562 cells (Supplemental Fig. 3f). Gene expression data from K562-*KDM6A*-KO cells revealed downregulation of allograft rejection and immune regulatory pathways, while drug transporters and *EZH2*-targets were among the most upregulated genes (Supplemental Fig. 4). Interestingly, *KDM6A* loss has been reported to enhance tumorigenicity of MM cells through unopposed *EZH2* activity [14]. We also reanalyzed previously published [13] gene expression data of K562 with *KDM6A* knockdown, and the antigen processing and presentation pathway was similarly among the most downregulated pathways in *KDM6A* knockdown cells.

In conclusion, our study provides novel insights of the potential impact of somatic mutations detected at diagnosis on the outcomes of treatment discontinuation in CML. The detection of mutations at the diagnosis can potentially contribute to the choice of frontline TKI, to negate the effect of mutations on the clinical outcome. Accordingly, recent studies have shown that the frontline use of second-generation TKIs potentially overcomes the negative impact of epigenetic modifier gene mutations on CML CP patients’ treatment response [8]. Our findings also suggest a potential link between some of the detected mutations, e.g., *KDM6A* mutations, and impaired immune responses in CML. Such mutations occur, however, in a small number of relapsed patients and cannot explain all relapses. Thus, characterization of larger patient groups is needed to increase understanding of the potential role of mutations in modulating CML immune responses. To date, genetic screening is not included in the current CML guidelines, despite recent evidence of somatic mutations contributing to treatment outcomes. Further studies are warranted to investigate the potential predictive value of genetic data as a biomarker for relapse after TKI stop, which would enable better selection of eligible patients for treatment discontinuation.

## Supplementary information

Supplemental data

Supplemental table 2
