# Supplementary material for: Epigenetic modifier gene mutations in chronic myeloid leukemia (CML) at diagnosis are associated with risk of relapse upon treatment discontinuation
Source: Blood Cancer J. 2022 Apr 20;12(4):69. doi: 10.1038/s41408-022-00667-9 (PMC9021312; doi:10.1038/s41408-022-00667-9)
Supplement: Supplementary file 1 — Supplemental data [file 41408_2022_667_MOESM1_ESM.docx]

***Epigenetic Modifier Gene Mutations in Chronic Myeloid Leukemia (CML) at Diagnosis Are Associated with Risk of Relapse upon Treatment Discontinuation***

Shady Adnan Awad^1,2,3,4^, Oscar Brück^1,2,3^, Naranie Shanmuganathan^5,6,7,8,9^, Timo Jarvinen^1,2^, Hanna Lähteenmäki^1,2^, Jay Klievink^1,2^, Hazem Ibrahim^10^, Soili Kytölä^11^, Perttu Koskenvesa^1^, Timothy P Hughes^5,7,9^, Susan Branford^6,7,8,9^, Matti Kankainen^1,2,3^, Satu Mustjoki^1,2,3^

^1^Hematology Research Unit Helsinki, University of Helsinki and Helsinki University Hospital Comprehensive Cancer Center, Helsinki, Finland, ^2^Translational Immunology Research Program and Department of Clinical Chemistry and Hematology, University of Helsinki, Helsinki, Finland, ^3^iCAN Digital Precision Cancer Medicine Flagship, Helsinki, Finland, ^4^Clinical Pathology Department, National Cancer Institute, Cairo University, Egypt, ^5^Department of Haematology, Royal Adelaide Hospital and SA Pathology, Adelaide, South Australia, ^6^Department of Genetics and Molecular Pathology and Centre for Cancer Biology, SA Pathology, Adelaide, South Australia, ^7^Precision Medicine Theme, South Australian Health and Medical Research Institute (SAHMRI), Adelaide, South Australia, ^8^School of Pharmacy and Medical Science, University of South Australia, Adelaide, South Australia, and ^9^School of Medicine, University of Adelaide, Adelaide, South Australia, Australia, ^10^Stem Cells and Metabolism Research Program, Faculty of Medicine, University of Helsinki, Helsinki, Finland, ^11^HUS Diagnostic Center, HUSLAB, Helsinki University Hospital, University of Helsinki, Helsinki, Finland

**Short title:** Somatic mutations and TKI discontinuation in CML

**Corresponding author**s

Prof. Satu Mustjoki

Hematology Research Unit Helsinki, University of Helsinki and Helsinki University Hospital Comprehensive Cancer Center, Haartmaninkatu 8, P.O. Box 700, FIN-00290 Helsinki, Finland, Tel +358 9 471 71898, Fax +358 9 471 71897, e-mail: [satu.mustjoki@helsinki.fi](mailto:satu.mustjoki@helsinki.fi)

Dr. Shady Adnan-Awad

Hematology Research Unit Helsinki, University of Helsinki and Helsinki University Hospital Comprehensive Cancer Center, Haartmaninkatu 8, P.O. Box 700, FIN-00290 Helsinki, Finland, email: [shady.awad@helsinki.fi](mailto:shady.awad@helsinki.fi)

**Supplemental Tables**

**Supplemental table 1: Characteristics of CML patient cohorts**

|  | **Cohort-1 (n=32)** | **Cohort-2 (n=15)** | **Patient**  **Cohort (n=47)** |
| --- | --- | --- | --- |
| **Age, median (range) years** | 50 (29-76) | 52 (32-78) | 52 (29-78) |
| **Gender, male: female** (%) | 23:9 (72:28) | 9:6 (60:40) | 32:15 (68:32) |
| **Frontline TKI,  imatinib: 2G-TKI** (%) | 21:11 (66:34) | 15:0  (100:0) | 36:11  (77:23) |
| **TKI Switch (%)*** | 3/21 (14%) | 4/15 (27%) | 7/36 (19%) |
| **TKI + IFN treatment (%)**** | 6 (19%) | _ | 6/32 (19%) |
| **Achievement of CCyR at 3 months, yes: no** (%)******* | 19:9 (68:32) | 11:4 (73:27) | 30:13 (70:30) |
| **On-TKI time prior to stop median (range) months** | 59 (18-124) | 67 (38-130) | 63 (18-130) |
| **Time of MR^4^ prior to stop median (range) months** | 36 (3-108) | 55 (31-85) | 44 (3-108) |
| **Follow-up time of TFR patients median (range) months** | 72 (60-138) | 49 (12-83) | 71 (12-138) |

CCyR: complete cytogenetic response, MR^4^: molecular response with reduction of *BCR-ABL1* level ≤ 0.01% on the International Scale. * TKI switch (referred to switch from imatinib to 2G-TKI) occurred in 7 of 36 frontline imatinib treated patients due to intolerance, including 2 patients who switched to dasatinib and 5 patients to nilotinib. ** TKI + IFN treatment was used in cohort-1 for 6 patients (5 imatinib treated and 1 dasatinib treated). *** the assessment of cytogenetic response at 3 months was available for 43 of 47 patients. We have used the achievement of CCyR (equivalent to 1% IS) at 3 months as an indicator of rapid deep response. All of the assessed patients, however, have achieved early molecular response milestone (<10% IS) at 3 months.

**Supplemental table 2: Genes included in the applied sequencing panels** (a separate datasheet).

|  | **TFR  (n=17)** | **Relapse (n=30)** | **Univariable analysis***** *P value* | **Multivariable analysis****** | | |
| --- | --- | --- | --- | --- | --- | --- |
|  |  |  |  | Log.reg.  *P value*  odd ratio | Cox reg.  *P value*  odd ratio | Bootstrap  *P value*  odd ratio |
| **Age, median (range) years** | 51.8 (32-71) | 51.9 (29-78) | 0.99 | _ | _ | _ |
| **Frontline TKI** imatinib 2G-TKI | 14 (82%) 3 (18%) | 22 (73%) 8 (27%) | 0.70 | _ | _ | _ |
| **Risk scores (%)**  Sokal (L:M:H,%)  Hasford (L:M:H,%)**^†^**  EUTOS (L:H,%)**^†^** | 56:31:13  46:54:0  100:0 | 43:36:21  53:35:12  93:7 | 0.64  0.38  0.99 | _ | _ | _ |
| **CCyR at 3 months** yes no | 10 (59%) 7 (41%) | 21 (70%) 9 (30%) | 0.77 | **_** | **_** | **_** |
| **Baseline hematological parameters**^†^** NK cell counts CD8 T cell counts lymphocyte proportion | 0.49 (0.22-1.38) 0.52 (0.17-1.57) 36% (15-62) | 0.35 (0.09-0.79) 0.38 (0.09-0.76) 30% (12-44) | 0.27 0.29 0.21 | _ | _ | _ |
| **On-TKI time** ≥50 months < 50 months | 14 (82%) 3 (18%) | 17 (57%) 13 (43%) | **0.037** | **0.084**  **-1.77** | **0.356**  **-0.711** | 0.173 (0.02-0.55)  -0.65 (-2.58 -0.34) |
| **Time of MR^4^** ≥24 months <24 months | 13 (76%) 4 (24%) | 24 (80%) 6 (20%) | 0.39 | _ | _ | _ |
| **SNV mutation load^†^** | 2.21 (1.11-3.56) | 2.24 (1.07-4.04) | 0.93 | _ | _ | _ |
| **Epigenetic modifier  gene mutations** yes no | 1 (6%) 16 (94%) | 8 (27%) 22 (73%) | **0.041** | **0.113**  **1.76** | **0.027**  **2.54** | 0.459 (0.04-0.99)  8.099 (0.53-18.06) |
| **Cancer-associated  gene mutations** yes no | 2 (12%) 15 (88%) | 10 (33%) 20 (67%) | **0.052** | **0.118**  **1.31** | **0.037**  **2.29** | 0.341 (0.05-0.99)  3.346 (0.48-18.34) |

**Supplemental table 3: Univariable and multivariable analyses between non-relapsing (TFR) and relapsing patient groups**

Log.reg.: logistic regression, Cox reg.: Cox regression. * Risk scores calculated at diagnosis. Sokal risk score data were available for 44 patients, Hasford and EUTOS scores data were available for patients from cohort 1 (28 and 24 patients respectively), L: low risk, M: intermediate risk, H: high risk. Numbers are represented as percentage of total number in each group. **Baseline hematological parameters include measurements at the time of TKI stop. Many parameters were analyzed, including total leukocyte counts, absolute counts and proportions of lymphocytes, T-cells, B-cells, NK-cells, CD4+ T-cells, CD8+ T-cells and monocytes. Selected parameters (*P*<0.3) are shown in the table. *** Univariable analyses were done as t-test for numerical parameters and Chi-square test for categorical parameters. **** Multivariable analysis was performed using logistic regression analysis in R including the following parameters: on-TKI time, the presence of mutations in cancer associated and epigenetic modifier genes. Bootstrapping p-values and odd ratios are expressed in mean and 90% confidence interval (90% CI) values. **^†^** Data is only available for cohort 1.

**Supplemental Figures**

**Supplemental Figure 1**

**Supplemental figure 1. Mutations in diagnostic samples from CP-CML patients**

Stacked columns comparing the prevalence of mutations in a) cancer-associated genes and b) epigenetic modifier genes between relapse and TFR patient groups in cohort 1 (*p-values* were 0.16 and 0.19 respectively, Chi-square test), and in cohort 2 (c,d; *p-values* were 0.44 and 0.05 respectively, Chi-square test).

**Supplemental Figure 2**

**Supplemental figure 2. Relapse-free survival of 47 CML patients classified according to**

a) the presence of mutations in cancer-associated genes, b) response to treatment at 3 months on-TKI (achievement of CCyR or not), c) TKI treatment period prior to stop (shorter or longer than 50 months), d) combination of presence of mutations in cancer-associated genes and TKI treatment period prior to stop (shorter or longer than 50 months). Log rank analysis was used in survival analysis including one variable (a-c), and stratified log-rank analysis was used in >1 factor comparison (d).

**Supplemental Figure 3**

**Supplemental figure 3. KDM6A in CML cells.** a) Detection of BCR-ABL1 in sorted cell populations from CML patient at diagnosis (D) and remission (Rem) time point using DNA-based PCR. b) Western blot shows the effect of KDM6A-KO on histone (H3) acetylation and methylation at Lysine residue 27 (H3K27). As expected, KDM6A-KO was associated with reduced acetylation and increased methylation at H3K27 compared with control cells. c) Comparison of the drug sensitivity scores (DSS), measured with CTG, to imatinib, dasatinib and ponatinib between K562 control cells and K562-KDM6A-KO cells. No notable differences were found in association with KDM6A-KO. The experiment was done in duplicate. K562 KDM6A-KO cells are less sensitive to NK-cell cytotoxicity compared to K562-ctrl cells using either d) freshly isolated NK cells from healthy donor buffy coats (n=2) or e) expanded activated NK cells (n=2). Each of E:T ratio was done in triplicate and two separate K562-KDM6A-KO clones were tested. The viability was measured using luciferase assay as described in the supplementary methods. f) Cytotoxic activity of NK92 *KDM6A*-KO cells against K562 cells compared to control NK92 cells. Viability or cytotoxicity was measured using luciferase assay as described in the supplementary methods. Reads from K562-NK coculture wells were normalized to reads from wells containing only K562 cells. Control: ctrl, negative control: NC, non-significant: ns. **:*p<0.01*, ***: *p<0.001*, ****:*p<0.0001*.

**Supplemental Figure 4**

**Supplemental figure 4. Transcriptional changes associated with *KDM6A*-KO in K562 cells.**

a) Volcano plot of protein coding genes between K562-*KDM6A-KO* and K562-control cells. Each gene is represented by a black dot and significant differentially expressed genes (Q<0.05, Bayesian statistical test) are colored red. b) Depiction of molecular pathways with significant altered expression between K562-*KDM6A-KO* and K562-control cells using the top differentially expressed genes (*Q<0.05*, >1.5 log foldchange). The red bars indicate the upregulated pathways in K562-*KDM6A-KO* cells and the blue bars indicates the downregulated pathways.

**Supplemental methods**

**Patient materials**

Diagnostic samples used in this study were derived from 2 cohorts. Cohort 1 (Helsinki University Hospital, Finland) included 32 CML patients (20 relapse and 12 TFR) and Cohort 2 (monitored in South Australia) included 15 patients (10 relapse and 5 TFR). In cohort 1, twenty-five patients were included in the Euro-Ski study,(1) and seven patients have been included in other TKI stop trials.(2) Only the availability of sample from the time of diagnosis and later TKI discontinuation attempt were used as selection criteria for the study. All patients in the study had given written informed consent.

**Cell lines**

K562 and NK92 cell lines were obtained from the Deutsche Sammlung von Mikroorganismen und Zellkulturen GmbH (DSMZ). Experiments were performed within 20 passages after obtaining cells from DSMZ, or with authenticating cells using GenePrint10 System (Promega). Cell lines were tested negative for mycoplasma using the MycoAlert kit (Lonza). Cell lines were cultured in RPMI-1640 (Lonza) with 10% FBS, 2 mM L-glutamine (Lonza), and 100 U/ml penicillin and 100 μg/ml streptomycin (Gibco) (R10). For NK92 cell line R10 media was supplemented with 10 ng/mL recombinant human IL-2 (Peprotech). K562 cells were transduced to express luciferase (K562-Luc) as previously described.(3)

**NK cell isolation and expansion**

Primary human NK cells were isolated from peripheral blood mononuclear cells (PBMC) obtained by Ficoll-Paque separation from buffy coats provided by the Finnish Red Cross. Isolation of NK cells was performed using the human NK cell isolation kit (Miltenyi Biotec). Freshly isolated NK cells of three healthy donors were directly used in co-culture cytotoxicity experiments. To obtain consistently active NK cells, primary NK cells were expanded and activated as previously described.(4) Before using in cytotoxicity experiments, expanded NK cells were thawn and cultured overnight in R10 supplemented with 10 ng/mL recombinant human IL-2.

**Next generation sequencing and variant calling**

For cohort 1, targeted sequencing capture was performed using SeqCap EZ Comprehensive Cancer Design panel (Roche NimbleGen, Madison, WI, USA) comprising 578 cancer-associated genes for 25 samples and inhouse-designed custom cancer gene panel (Finnish Institute of Molecular Medicine) comprising 1853 cancer-associated genes for 7 samples. The inhouse designed panel is based on Twist Bioscience dsDNA probes and library preparation kits and includes all 578 genes covered by the Comprehensive Cancer Design panel. Sequencing libraries were prepared and subjected to 2×100-bp paired-end sequencing on NovaSeq instrument (Illumina, San Diego, CA, USA) according to the manufacturer's instructions. The mean coverage depth for samples in cohort 1 is 340x.

Variant calling and analysis of DNA sequencing data were performed as previously described.(5) Briefly, Trimmomatic software(6) was used to pre-process sequence data for low quality, adapter sequences, unique molecular identifier (UMI) and short read length. After pre-processing, paired-end reads were aligned, using BWA-MEM,(7) to human reference genome build 38 (EnsEMBL v82) and alignments were sorted by coordinate using the SortSAM. MarkDuplicate module of the Picard toolkit (Broad Institute) were used to mark PCR duplicates. Genome Analysis Toolkit (GATK) toolset(8) was used to call variants, applying the GATK somatic short variant best practice (version 3.5), supplemented with GATK4CalculateContamination, CollectSequencingArtifactMetrics, and FilterByOrientationBias tools. GATK resources were converted from GRCh37 to GRCh38 using CrossMap.(9) Variant calls were then normalized using bcftools.(10)

Filtration and annotation of variants were performed using the Annovar tool(11) against the RefGene database as previously described.(5) Variants other than those passing all MuTect2 filters were filtered. Variant data were further filtered by removing intronic variants, poorly covered and low-quality variants, as well as common SNPs as identified by ExAC, gnomAD exome, or gnomAD genome databases. To select potentially non-silent variants, the previous variant call set was filtered further by removing synonymous mutations and non-frameshift variants, variants with a total coverage <50, and those with minor allele frequency of >0.01% in Finnish ExAC and gnomAD databases. Variant selection from the samples prepared using the inhouse 1853 genes panel was further restricted to the 578 genes included in the Comprehensive Cancer Design panel, to make the data consistent. Finally, cancer associated mutations were picked by selecting mutation in epigenetic modifier genes and mutations with COSMIC(12) identifier in hematological cancers. All of the selected mutations have been confirmed by orthogonal sequencing method of the leukemic samples. Selected mutations were also confirmed as somatic, by sequencing control non-leukemic samples (such as sorted T-cells or remission samples).

For cohort 2, targeted sequencing was performed using an RNA-based inhouse designed hybridization capture method that targeted the exons of 130 genes relevant for myeloid and lymphoid leukemia.(13–15) The custom library of probes was designed using NimbleDesign software (Roche SeqCap EZ Prime Choice Probes ≤ 7Mb, hg19 Genome Build). Target locations were uploaded to the Roche custom design tool, NimbleDesign, to generate probes with lengths between 50 to 105 nucleotides. Libraries were prepared using the Illumina TruSeq Stranded Total RNA with ribosomal depletion using 1ug of DNAse treated RNA. Target regions were enriched using the custom oligo capture probes. 150bp paired-end sequencing was performed on an Illumina NextSeq 500 using the NextSeq 500/550 Mid Output Kit V2.5. The raw sequencing reads were aligned to the b37-decoy reference genome (Genome Analysis Toolkit (GATK) Resource bundle human_g1k_v37_decoy) using the STAR algorithm v2.7.2b and two pass mode for splice site definition. UMI-tools(16) were used for removing PCR duplicates. Variant calling was performed using the FreeBayes software v1.2.0.(17) Variants were annotated using an inhouse pipeline with customized filtering strategies to identify relevant variants. The clinical relevance and pathogenicity of variants was assessed using strict criteria as previously described.(13) All cancer-associated variants were verified using Sanger sequencing and were confirmed as somatic using Sanger sequencing and a corresponding remission sample (undetectable *BCR-ABL1* transcripts MR4.5, ≤0.0032% IS).

**Flowcytometry-based sample fractionation and amplicon sequencing**

For CML patient_2 with the KDM6A mutation identified at diagnosis, peripheral blood mononuclear cell (PMNC) samples were collected both at diagnosis and remission (i.e., molecular remission after resuming TKI treatment upon disease relapse) time points; to investigate the presence of the mutation in different cell types. Cells were stained for 15 minutes with surface markers CD3-APC (Cat# 555335 and CD56-PE (Cat#345812) from BD Biosciences (San Diego, CA, USA). The cells were then acquired and sorted using FACSAria II for T-lymphocytes (CD3+ CD56-), NK-cells (CD3- CD56+), NKT-cells (CD3+ CD56+) and myeloid/monocytic fraction (CD3- CD56-). DNA was extracted from each fraction and analyzed using amplicon sequencing. For sequencing, the following primers for *KDM6A* exon 18 (KDM6A-ex18-F*: 5`-TTGGCACATAGCTCAGGTTG-3`,* KDM6A-ex18-R*: 5`-CAAATACTTACTCTCAATGATTCCTG-3`*) were used. The sequencing was performed using paired end 300 bp (PE300) on MiSeq instrument (Illumina, San Diego, CA, USA). The KDM6A Q1058X mutation was identified in myeloid and NK cell fraction from diagnosis samples only but not from other fractions at either timepoints. For detection of BCR-ABL in sorted cell subsets, we applied DNA-based PCR amplification using a forward primer: TCCGCTGACCATCAAYAAGGA (*BCR, exon 13*) and a reverse primer: CACTCAGACCCTGAGGCT CAA (*ABL1, exon 2*).

**CRISPR/Cas9 KDM6A gene editing**

To knockout KDM6A gene, we designed sgRNA targeting exon 4 of human *KDM6A (*KDM6A-sgRNA-F*: 5`-GGTATGCAGATAATGCTGAA-3`,* KDM6A-sgRNA-R*:* *5`-TTCAGCATTATCTGCATACC-3`),* which was cloned in lentiCRISPRv2-GFP backbone plasmid (Addgene #52961). K562-Luc cells were then either lentivirally transduced for stable expression Cas9-sgRNA in the cells or transfected using Fugene HD transfection reagent (Promega) inducing transient expression of Cas9-sgRNA in the cells. Cells were sorted for GFP positivity using Sony SH800 cell sorter (Sony Biotechnology). For delivery of CRISPR/Cas9 to NK92 cells, cells were electroporated using Neon Transfection System (Thermo Fisher Scientific) using previously described parameters (2 pulses, 1^st^ pulse: 1650 V, 20 milliseconds and 2^nd^ pulse: 500 V, 100 milliseconds).(18) *KDM6A* knockout efficiency was assessed using capillary sequencing, using designed PCR primers for KDM6A exon 4 (KDM6A-ex4-f:*5`-*GGGGTTAGCCTAGATGCTGTTC-*3`*, KDM6A-ex4-R: *5`-*ATTGGCAATAATCTGCCCAAAACA-*3`*) and predicted effects were assessed using Synthego ICE CRISPR Analysis tool (v2). Successful knockout clones (K562-KDM6A-KO) were further expanded and used in cytotoxicity experiments. K562 cells that were similarly processed (transduced or transfected) and had unedited (i.e., wild type) *KDM6A* gene were selected and used as a control (K562-Luc-ctrl).

**NK cell cytotoxicity experiment**

For co-culture cytotoxicity experiments, K562-Luc-KDM6A-KO and K562-Luc-ctrl cells were plated as 20,000 cells/well on a 96 well plate together with fresh (n=2) or expanded (n=2) NK cells at the indicated effector: target (E:T) ratio in a total volume of 50ul. Each of E:T was done in triplicate. Plates were incubated at 37 °C and 5% CO2 for 6 h, after which target cell viability was measured by dispensing 50 µl ONE-Glo reagent to each well and reading luminescence with a Pherastar FS plate reader. Similar settings were used to test the cytotoxic activity of NK92-KDM6A-KO and NK92-ctrl cells against K562-Luc cells. For flow cytometry characterization of NK cells in coculture cytotoxicity assay, the following antibodies were used: mouse anti-human CD3 APC (Cat#340440), mouse anti-human CD56 Pe-Cy7 (Cat#557747), anti-human CD16 PE (Cat#561313) V450 Mouse Anti-Human CD107a (Cat#561345) and 7-AAD stain (Cat#559925). All antibodies were purchased from BD Bioscience.

**Western blot**

To investigate the effect of KDM6A-KO on histone 3 (H3) acetylation and methylation, the following antibodies were used: Histone H3 rabbit antibody (Cat# 9715S), Tri-Methyl-Histone H3 (Lys27) (C36B11) rabbit mb (Cat#9733), Acetyl-Histone H3 (Lys27) (D5E4) rabbit mb (Cat#8173) in addition to β-Actin (8H10D10) mouse mAb (Cat#3700). All antibodies were purchased from Cell Signaling Technology.

**Pathway enrichment analysis**

Analysis of the enriched pathways in association with KDM6A knockout in K562 cells was performed on expression data from a previously published study,(19) using Enrichr tool.(20,21)

**Statistical analysis**

Two-tailed Student t-test and Fisher exact chi square test were computed using GraphPad Prism 8 software. For co-culture cytotoxicity experiments, comparisons were performed at all E:T ratios using multiple unpaired t-tests and nested t-test using GraphPad Prism 8 software. For the relapse-free survival and hazard ratio analysis, log-rank (Mantel-Cox) and stratified log-rank tests were applied using GraphPad Prism 8 software. For multivariable analysis, logistic and Cox regression analyses were performed in R using ‘janitor’ and ‘survival’ packages. For validation of the multivariable analysis results, we used Bootstrapping regression function in R with 50 random sample replacement.

**Reference**

1. Saussele S, Richter J, Guilhot J, Gruber FX, Hjorth-Hansen H, Almeida A, et al. Discontinuation of tyrosine kinase inhibitor therapy in chronic myeloid leukaemia (EURO-SKI): a prespecified interim analysis of a prospective, multicentre, non-randomised, trial. Lancet Oncol. 2018;19(6):747–57.

2. Koskenvesa P, Kreutzman A, Rohon P, Pihlman M, Vakkila E, Räsänen A, et al. Imatinib and pegylated IFN-α2b discontinuation in first-line chronic myeloid leukemia patients following a major molecular response. Eur J Haematol. 2014;92(5):413–20.

3. Dufva O, Koski J, Maliniemi P, Ianevski A, Klievink J, Leitner J, et al. Integrated drug profiling and CRISPR screening identify essential pathways for CAR T-cell cytotoxicity. Blood. 2020 Feb 27;135(9):597–609.

4. Denman CJ, Senyukov VV, Somanchi SS, Phatarpekar PV, Kopp LM, Johnson JL, et al. Membrane-bound IL-21 promotes sustained ex vivo proliferation of human natural killer cells. PLoS One. 2012;7(1):e30264.

5. Adnan Awad S, Kankainen M, Ojala T, Koskenvesa P, Eldfors S, Ghimire B, et al. Mutation accumulation in cancer genes relates to nonoptimal outcome in chronic myeloid leukemia. Blood Adv. 2020 Feb 11;4(3):546–59.

6. Bolger AM, Lohse M, Usadel B. Trimmomatic: a flexible trimmer for Illumina sequence data. Bioinformatics. 2014 Aug 1;30(15):2114–20.

7. Li H. Aligning sequence reads, clone sequences and assembly contigs with BWA-MEM. arXiv:13033997 [q-bio]. 2013 Mar 16;http://arxiv.org/abs/1303.3997.

8. McKenna A, Hanna M, Banks E, Sivachenko A, Cibulskis K, Kernytsky A, et al. The Genome Analysis Toolkit: A MapReduce framework for analyzing next-generation DNA sequencing data. Genome Res. 2010 Sep;20(9):1297–303.

9. Zhao H, Sun Z, Wang J, Huang H, Kocher J-P, Wang L. CrossMap: a versatile tool for coordinate conversion between genome assemblies. Bioinformatics. 2014 Apr 1;30(7):1006–7.

10. Li H. BFC: correcting Illumina sequencing errors. Bioinformatics. 2015 Sep 1;31(17):2885–7.

11. Wang K, Li M, Hakonarson H. ANNOVAR: functional annotation of genetic variants from high-throughput sequencing data. Nucleic Acids Res. 2010 Sep;38(16):e164.

12. Forbes SA, Beare D, Boutselakis H, Bamford S, Bindal N, Tate J, et al. COSMIC: somatic cancer genetics at high-resolution. Nucleic Acids Res. 2017 Jan 4;45(D1):D777–83.

13. Branford S, Wang P, Yeung DT, Thomson D, Purins A, Wadham C, et al. Integrative genomic analysis reveals cancer-associated mutations at diagnosis of CML in patients with high-risk disease. Blood. 2018 30;132(9):948–61.

14. Iacobucci I, Mullighan CG. Genetic Basis of Acute Lymphoblastic Leukemia. J Clin Oncol. 2017 Mar 20;35(9):975–83.

15. Lindsley RC, Mar BG, Mazzola E, Grauman PV, Shareef S, Allen SL, et al. Acute myeloid leukemia ontogeny is defined by distinct somatic mutations. Blood. 2015 Feb 26;125(9):1367–76.

16. Smith T, Heger A, Sudbery I. UMI-tools: modeling sequencing errors in Unique Molecular Identifiers to improve quantification accuracy. Genome Res. 2017 Mar;27(3):491–9.

17. Garrison E, Marth G. Haplotype-based variant detection from short-read sequencing. arXiv:12073907 [q-bio] [Internet]. 2012 Jul 20; Available from: http://arxiv.org/abs/1207.3907

18. Ingegnere T, Mariotti FR, Pelosi A, Quintarelli C, De Angelis B, Tumino N, et al. Human CAR NK Cells: A New Non-viral Method Allowing High Efficient Transfection and Strong Tumor Cell Killing. Front Immunol [Internet].2019;10. https://www.frontiersin.org/articles/10.3389/fimmu.2019.00957/full

19. Stief SM, Hanneforth A-L, Weser S, Mattes R, Carlet M, Liu W-H, et al. Loss of KDM6A confers drug resistance in acute myeloid leukemia. Leukemia. 2020 Jan;34(1):50–62.

20. Chen EY, Tan CM, Kou Y, Duan Q, Wang Z, Meirelles GV, et al. Enrichr: interactive and collaborative HTML5 gene list enrichment analysis tool. BMC Bioinformatics. 2013 Apr 15;14:128.

21. Kuleshov MV, Jones MR, Rouillard AD, Fernandez NF, Duan Q, Wang Z, et al. Enrichr: a comprehensive gene set enrichment analysis web server 2016 update. Nucleic Acids Res. 2016 Jul 8;44(W1):W90-97.
